# Correction to: NCR13 peptide protects soybean against *Cercospora sojina* using multi-faceted modes of action and additive interaction with chemical fungicides

**DOI:** 10.1093/jxb/erag301

**Published:** 2026-08-10

This is a correction to:

Ambika Pokhrel, Vishnu Sukumari Nath, James Godwin, Raviraj Kalunke, Meenakshi Tetorya, Kirk J Czymmek, Dilip M Shah, NCR13 peptide protects soybean against *Cercospora sojina* using multi-faceted modes of action and additive interaction with chemical fungicides, *Journal of Experimental Botany*, Volume 76, Issue 19, 13 October 2025, Pages 6624–6641, https://doi.org/10.1093/jxb/eraf446

In October 2025, the authors contacted the journal to request some corrections to the methodology and results of this article.

Peptide concentrations were quantified using the Bicinchoninic Acid (BCA) assay. However, after publication the authors found that the BCA assay significantly underestimates the concentration of the peptide NCR13_PFV1 due to its unique physicochemical properties that interfere with an accurate quantification by BCA. The authors therefore performed amino acid analysis (AAA) for NCR13_PFV1 and NCR13_PFV2. Based on the AA analysis, the minimum inhibitory concentration (MIC) of these two peptides against *Cercospora sojina* were corrected as follows: The MIC against *C. sojina* Cs350 (fungicide resistant isolate) is 1.5 µM for NCR13_PFV1 and 3 µM for NCR13_PFV2. The MIC against *C. sojina* Cs320 (fungicide sensitive isolate) is 0.75 µM for NCR13_PFV1 and 1.5 µM for NCR13_PFV2. The MIC against *Botrytis cinerea* is 0.375 µM for NCR13_PFV1 and 0.75 µM for NCR13_PFV2. These findings were consistent across two independent peptide batches and replicated in *in vitro* antifungal assays. The peptide concentrations were corrected throughout the text and in Figures 1, 3, 4, 5, 7, 8, S1, S5, S6, S7, S8.

A paragraph describing the AAA was added to the Material and Methods section.

In Fig. 1A the MIC data for fungicide sensitive isolates Cs324 and Cs752 as well as fungicide resistant isolates Cs326 and Cs673 were removed. The assay could not be repeated with these cultures as they are not actively maintained in the authors’ laboratory. These isolates are not central to the study and removal of the data does not change the overall conclusions.

The heme binding assay presented in Fig. 7A and Fig. 7B was repeated with equal concentrations of 5 μM for all peptides. The results remained consistent, confirming that NCR13 does not exhibit heme binding activity. The figure was revised accordingly.

The RNAseq analysis previously reported gene expression changes in *C. sojina* and *B. cinerea* after treatment with equal concentrations of both NCR13_PFV1 and NCR13_PFV2, specifically 0.187 µM for *C. sojina* and 0.09 µM for *B. cinerea*. Since the new MIC of NCR13_PFV1 and NCR13_PFV2 changed 8-fold and 2-fold, respectively, the comparison has become inconsistent. The RNAseq data for NCR13_PFV2 was therefore removed by omitting previous Figure S5 and revising previous Figures S6B and S6D. The numbering of all supplementary figures was changed accordingly. Since NCR13_PFV2 is not the main focus of the study, this revision does not affect the interpretation or conclusions of the RNAseq findings.

The authors confirm that the overall interpretation and conclusions of the study remain unchanged. The biological efficacy and comparative performance of the peptides are still valid and supported by the revised data. The editor and a reviewer have reviewed the revised data, approved the corrections, and are satisfied that the changes do not affect the conclusions of the article. The authors sincerely apologize for any inconvenience this may have caused.

