# Supplementary material for: NCR13 peptide protects soybean against Cercospora sojina using multi-faceted modes of action and additive interaction with chemical fungicides
Source: J Exp Bot. 2025 Oct 13;77(19):6624–41. doi: 10.1093/jxb/eraf446 (PMC13672995; doi:10.1093/jxb/eraf446)
Supplement: Supplementary file 1 [file eraf446_supplementary_data.zip › Supplementary_Figures_corrected.pdf]

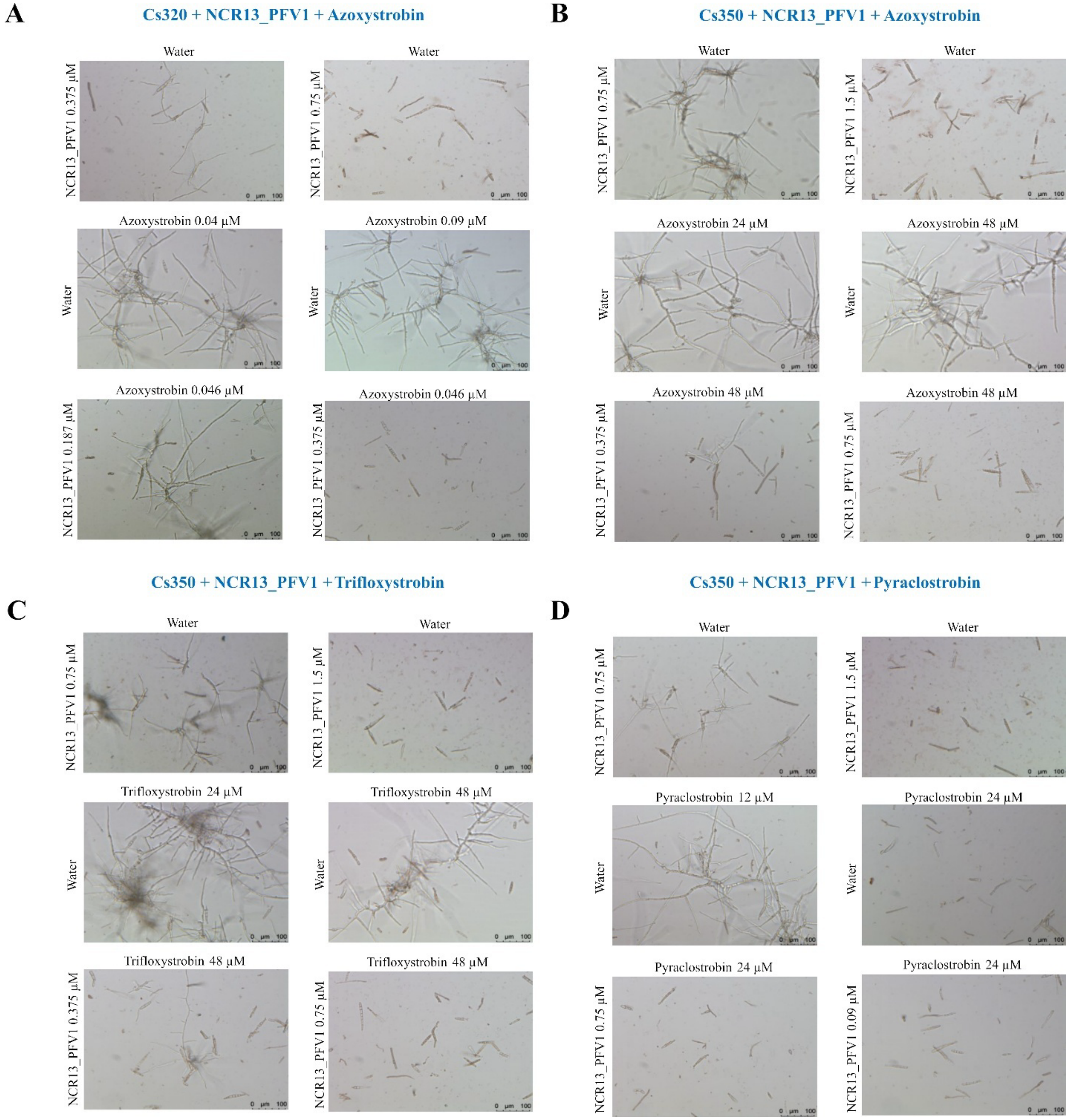

Fig. S1: NCR13_PFV1 exhibits additive antifungal activity with quinone outside inhibitor (QoI) fungicides against *C. sojina*. (A) The light microscopy images of fungicide sensitive *C. sojina* isolate Cs320 in various combination of NCR13_PFV1 and Azoxystrobin. Azoxystrobin was tested in the range of 0.01 to 0.09 µM and NCR13_PFV1 was tested in the range of 0.04 to 0.75 µM. The images at MIC and below MIC are shown if applicable. **(B) – (D)** The light microscopy images of fungicide resistant *C. sojina* isolate Cs350 in various combination of NCR13_PFV1 with Azoxystrobin, Trifloxystrobin, and Pyraclostrobin respectively. Fungicides were tested in the range of 1.5 to 48 µM and NCR13_PFV1 was tested in the range of 0.09 to 1.5 µM. The images at MIC and below MIC are shown if applicable.

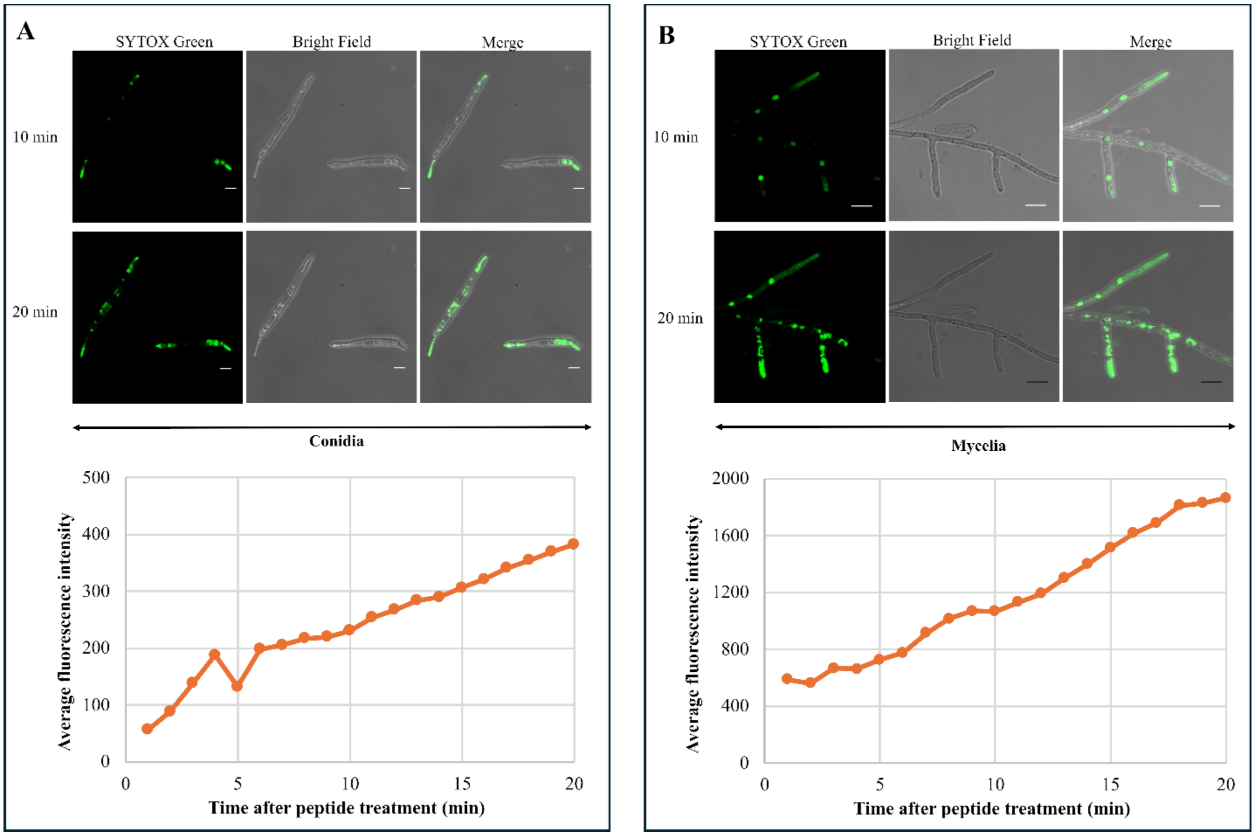

Fig. S2: NCR13_PFV1 permeabilizes the plasma membrane in *C. sojina*. Confocal microscopy images of SYTOX Green (SG) uptake in *C. sojina* and their average fluorescence intensity in **(A)** conidia and **(B)** mycelia treated with 1.5 µM of NCR13_PFV1. Time lapse confocal microscopy was performed in *C. sojina* and representative images at 10 and 20 minutes after peptide treatment are shown. Scale bars = 10 µm.

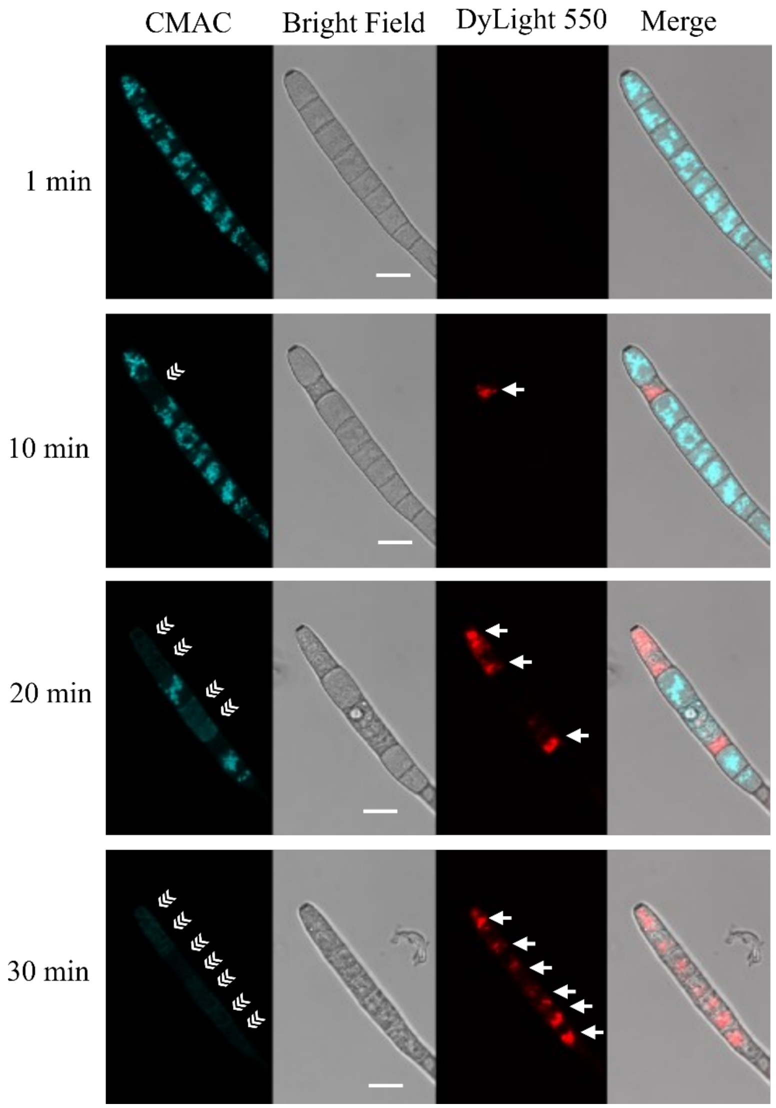

Fig. S3: NCR13_PFV1 does not localize in vacuoles in *C. sojina* Cs350. Timelapse confocal microscopy images of *C. sojina* showing the internalization of 1.5 µM DyLight550 labelled NCR13_PFV1. Vacuole specific dye CMAC was used to stain *C. sojina* vacuoles. Triple white arrows indicate the bursting of vacuole, and single white arrows indicate the aggregation of peptide. Scale bars = 10 µm.

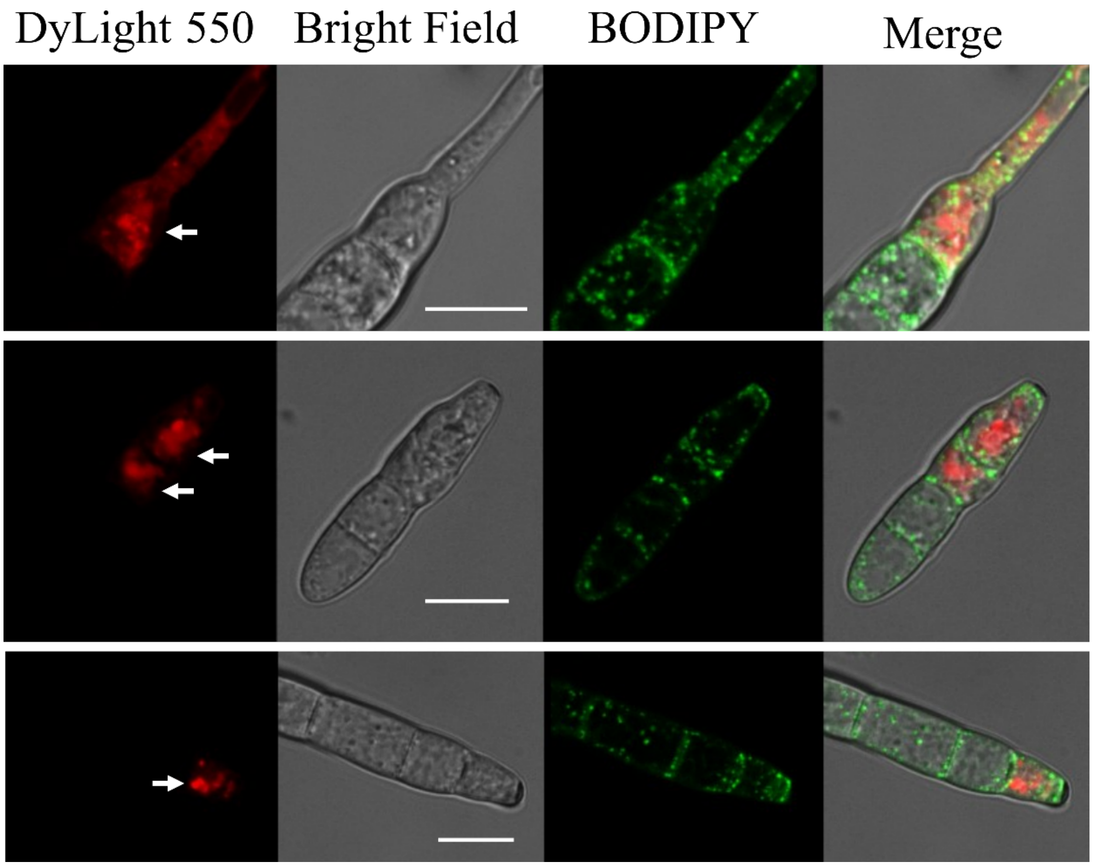

Fig. S4: NCR13_PFV1 does not localize in lipids in *C. sojina* Cs350. Timelapse confocal microscopy images of *C. sojina* showing the internalization of 1.5 µM DyLight550 labelled NCR13_PFV1. Lipid specific dye BODIPY was used to stain *C. sojina* lipids. Single white arrows indicate the aggregation of peptide. Scale bars = 10 µm.

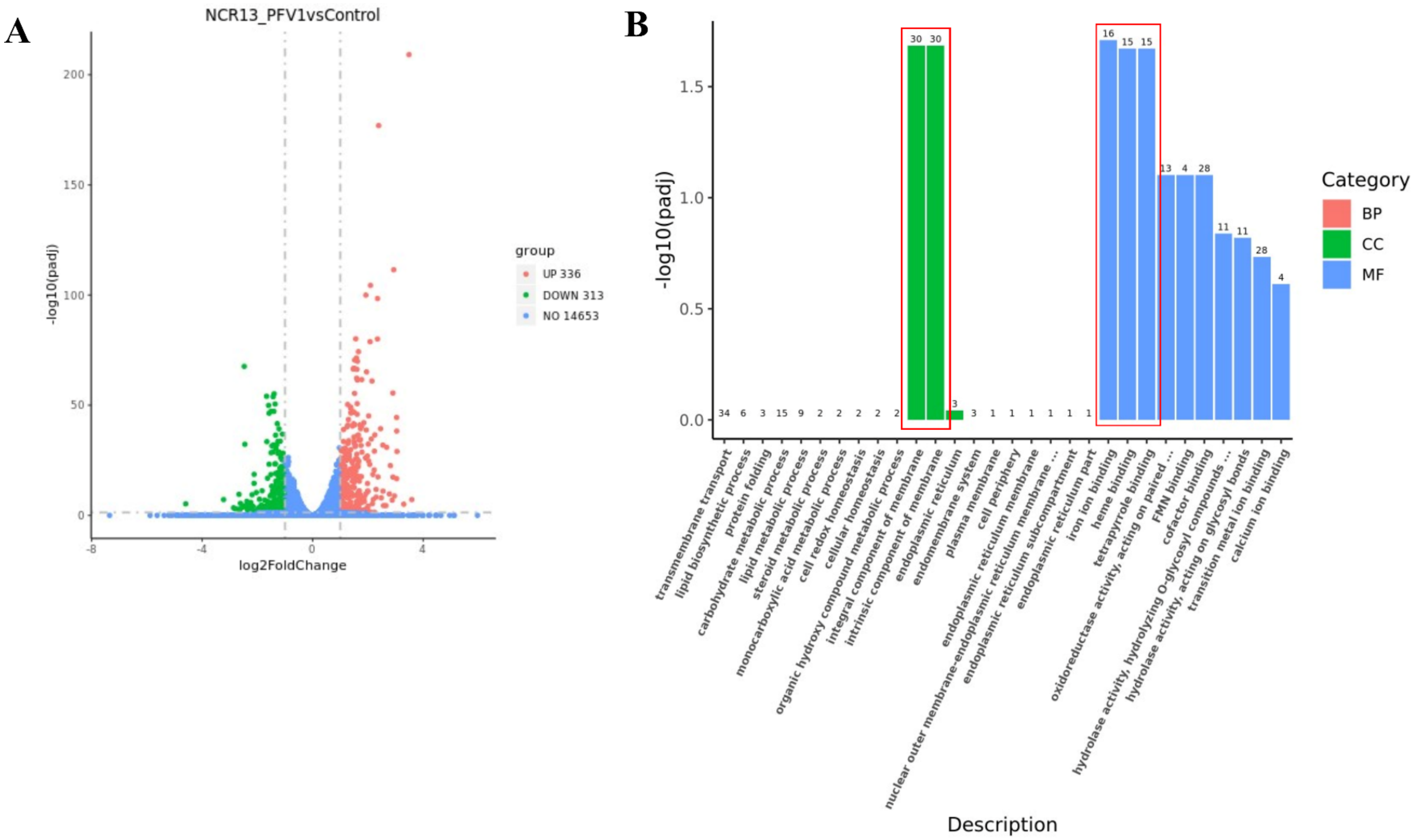

Fig. S5: NCR13_PFV1 induces genes encoding heme binding proteins in *B cinerea*. (A) Volcano plot showing the number of differentially expressed genes (DEGs) in *B. cinerea* after 30 min of NCR13_PFV1 treatment when compared to the no peptide control at 0 min. **(B)** Bar graph showing the top ten GO terms enriched in each category (BP-Biological Process, CC-Cellular Component, and MF-Molecular Function) at 30 mins after NCR13_PFV1 treatment in *B. cinerea*. The significantly enriched GO terms at p-value≤0.05 are highlighted in red rectangular box.

A

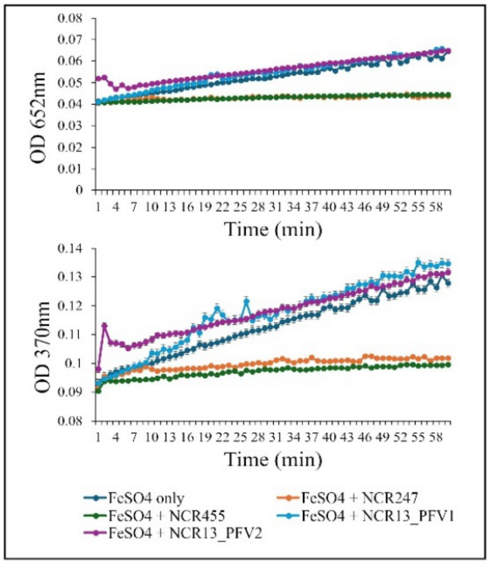

B

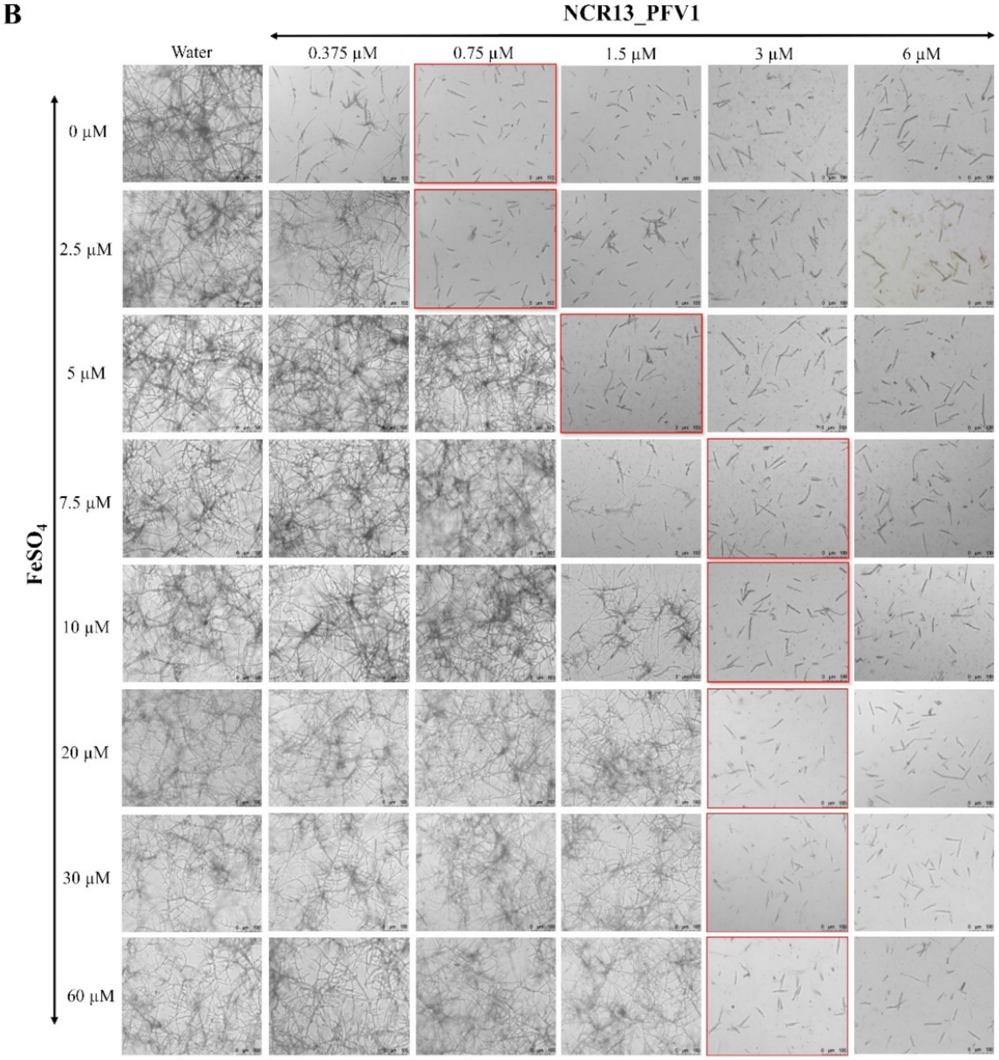

C

Iron (FeSO4) concentration (µM)	MIC against *C. sojina* Cs350 (µM)		
	NCR13_PFV2	NCR13_V7	NCR13_V9
**5 (Complete SFM)**	**3**	**6**	**3**
20	6	12	6

D

	Zinc ($ZnSO_4$) concentration (µM)	MIC of NCR13_PFV1 against *C. sojina* Cs350 (µM)
**Complete SFM**	**0.5**	**1.5**
	2.5	1.5
increased	5	1.5
	10	1.5

Fig. S6: *C. sojina* Cs350 exhibits iron mediated tolerance to NCR13 and its variants. (A) Peroxidase activity of $FeSO_4$ (75 µM) and peptides, NCR247, NCR455, NCR13_PFV1, and NCR13_PFV2 on a chromogenic substrate, 3,3′,5,5′-tetramethylbenzidine (TMB), measured by absorption at 370 nm and 652 nm. **(B)** Light microscopy images of *C. sojina* at various concentration of NCR13_PFV1 and $FeSO_4$. The images highlighted in red indicate the MIC level of *C. sojina* at the indicated concentration of NCR13_PFV1 and $FeSO_4$. **(C)** MIC of NCR13 variants against *C. sojina* at various concentration of iron ($FeSO_4$). **(D)** MIC of NCR13_PFV1 against *C. sojina* at various concentration of zinc ($ZnSO_4$).

A

	Manganese ($MnSO_4$) concentration (µM)	MIC of NCR13_PFV1 against *C. sojina* Cs350 (µM)
Complete SFM	0.1	1.5
	0.5	1.5
increased	1	1.5
	2	1.5

B

	Calcium ($CaCl_2$) concentration (µM)	MIC of NCR13_PFV1 against *C. sojina* Cs350 (µM)
Complete SFM	0	1.5
	10	1.5
increased	20	1.5
	200	12
	2000	>12

Fig. S7: Effect of NCR13_PFV1 on *C. sojina* Cs350 at elevated concentrations of manganese and calcium. **(A)** MIC of NCR13_PFV1 against *C. sojina* at various concentration of manganese ($MnSO_4$). **(B)** MIC of NCR13_PFV1 against *C. sojina* at various concentration of calcium ($CaCl_2$).

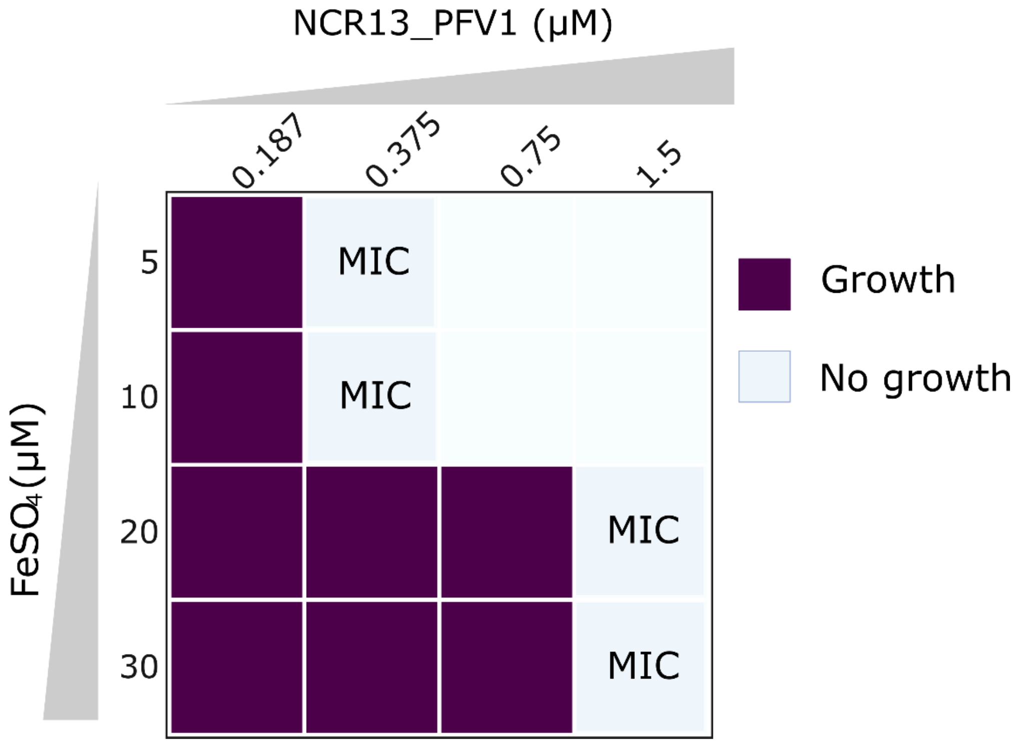

Fig. S8: *B. cinerea* exhibits iron mediated tolerance to NCR13_PFV1. MIC of NCR13_PFV1 against *B. cinerea* at various concentration of iron ($FeSO_4$).
